# A Software Architecture for the Industrial Internet of Things—A Conceptual Model

**DOI:** 10.3390/s20195603

**Published:** 2020-09-30

**Authors:** Ioan Ungurean, Nicoleta Cristina Gaitan

**Affiliations:** 1 Faculty of Electrical Engineering and Computer Science, Stefan cel Mare University of Suceava, 720229 Suceava, Romania; 2 MANSiD Integrated Center, Stefan cel Mare University, 720229 Suceava, Romania

**Keywords:** Internet of Things, Industrial Internet of Things, software architectures, fieldbuses, fog/edge computing, system on chip

## Abstract

The Internet of Things (IoT) is an emerging concept that has revolutionized the use of new technologies in everyday life. The economic impact of IoT becoming very important, and it began to be used in the industrial environment under the name of the Industrial Internet of Things (IIoT) concept, which is a sub-domain of IoT. The IIoT changes the way industrial processes are controlled and monitored, increasing operating efficiency. This article proposes a software architecture for IIoT that has a low degree of abstraction compared to the reference architectures presented in the literature. The architecture is organized on four-layer and it integrates the latest concepts related to fog and edge computing. These concepts are activated through the use of fog/edge/gateway nodes, where the processing of data acquired from things is performed and it is the place where things interact with each other in the virtual environment. The main contributions of this paper are the proposal and description of a complete IIoT software architecture, the use of a unified address space, and the use of the computing platform based on SoC (System on Chip) with specialized co-processors in order to be able to execute in real-time certain time-critical operations specific to the industrial environment.

## 1. Introduction

The Internet of Things (IoT) [1,2] is an emerging concept that changes the interactions of people with things in everyday life. IoT [3] allows the connection of ubiquitous objects/things to the Internet in order to provide innovative services that can lead to time- and money-saving in the daily activity of the individuals and that can increase the quality of life [4].

The IoT concept is applied in a wide variety of applications such as smart building [5,6] smart transport [7], smart cities [8], smart healthcare [9,10], and smart living with the aim to provide new services and an efficiency of the operating costs. In the design and development of IoT applications, existing technologies can be used to activate the IoT concept, but new technologies can be developed to help develop the specific applications. Initially, the IoT concept uses only wireless technologies for connection of the things to the Internet but now it can use any available technologies, wired and wireless. In fact, IoT can reuse wired or wireless communication technologies that have been used in other types of application in which various devices can connect to a computing system directly or through a gateway.

The things/objects form everyday life are brought into the virtual environment through sensors or other methods of acquiring real-data from their environment, such as human–machine interfaces. Acquired data or virtual things are transmitted to computing platforms where they are processed, they interact between them, and decisions are made, decisions that can be transmitted to the execution elements located in the environment. Basically, the things interact with each other in the virtual environment, opening the way to a wide variety of applications, from monitoring, identification, tracking, metering, resource management, etc. [11]. IoT is an evolution of the Internet from the Internet of People to the Internet of Things and further to the Internet of Everything.

All these things connected to the Internet generate a large volume of data and sending this data directly to the cloud is costly in terms of bandwidth and time of response to events. It is estimated that the number of IoT devices will be about 41.6 billion and will generate 79.4 zettabytes in 2025 [12] with an annual growth rate of 28.7% from 2018 to 2025. For this reason, it is not efficient to transfer this data directly to the cloud because they would use a considerable bandwidth. In order to address this issue, the concept of edge/fog computing has been defined, through which these data can be processed much closer to their source and some cloud services are brought to the edge of the network.

The differences between fog and edge computing concepts are given in the way the data are processed. For data processing, fog computing uses the interconnection between nodes while edge computing is performed on isolated nodes, closer to where the data is generated [13].

A sub-domain of IoT is the Industrial Internet of Things (IIoT) [14,15] that includes the industrial and machine-to-machine (M2M) communication technologies used in a smart factory or in the automation fields. In addition to IoT, the IIoT [4] has new challenges such as latency constraints, network bandwidth constrains, resource-constrains devices, reliability, uninterrupted service without Internet access, and new security challenges that are specific to the industrial field. Information services provided throughout the IIoT are still in their early stage [16]. Legacy systems in manufacturing with proprietary data and protocols are seen as one of the major existing challenges in the development of IIoT solutions [16]. The IIoT consists of sensor networks (industrial fieldbuses), actuators, robots, machines, appliances, business processes, and personnel [17]. All of these elements are used to achieve an efficient and intelligent manufacturing process. Industry 4.0 is a sub-domain of IIoT and refers to the integration of the IoT concept into smart manufacturing. The term of Industry 4.0 was proposed by the German government to define the fourth industrial revolution [18].

A definition of the IIoT is provided in [19], where IIoT is defined as “the network of intelligent and highly connected industrial components that are deployed to achieve high production rate with reduced operational costs through real-time monitoring, efficient management and controlling of industrial processes, assets and operational time”.

The IIoT market has a high economic potential. It is estimated that the IIoT market will reach the value of $263.4 billion in 2027 despite the crisis from 2020 with an annual growth of 16.7% from 2019 to 2027 [20]. The IIoT caught the attention of the academic, government, and industrial environment due to the benefits it can provide.

In the industrial environment, sensors used include thermocouples (TC), resistance temperature detectors (RTDs), or other types of transducer (for example pressure, flow, liquid level transducers). Usually, these sensors/transducers are connected to the devices with digital/analog inputs, which in turn are connected to an industrial communication bus called a fieldbus. Furthermore, the transducers can be connected directly to the fieldbuses. These devices can have low complexity in the sense that they can read the values from the inputs and transmit them on the fieldbuses, or devices that can have high complexity and they can make different decisions based on the inputs, such are the programmable logic controllers (PLCs). These devices can also have analog/digital outputs and can be used to control different actuators. There can be actuators connected directly to fieldbuses to receive commands [21]. In the industrial environment, there is a wide variety of devices that combine the features presented so far.

The industrial environment is a special environment where we can find many sources of interference in wireless communication systems. Also, wireless communication is not suitable for the development of control systems with real-time requirements. For this reason, wire communication systems such as EtherCAT, CANOpen, Profinet, Profibus, Modbus, and AS-I fieldbuses [21] are mainly used, but there are also wireless fieldbuses such as WirelessHART or the new technologies based on the 5G [22,23], such as Narrowband IoT or Long-Term Evolution (LTE) Cat-M.

In the design and development of IIoT solutions, the main issues and challenges are:

- The largest number and great diversity of fieldbuses and devices used in the industrial environment;
- The compliance with real-time requirements specific to the industrial applications;
- Interoperability between fieldbuses and devices;
- Interoperability between IIoT systems.

In the specialized literature, several reference architectures for IoT and IIoT are proposed, but these are abstract models which do not deal with how to integrate things from the industrial environment, especially as in this environment are used communication systems (fieldbuses) with specific capabilities such as real-time monitoring and control of time-critical operations. Practical examples are also presented in the specialized literature, but these solutions are designed and developed for a certain type of network and a small number of device types.

This paper proposes a software architecture for IIoT that integrates the concepts of fog/edge computing and fieldbuses specific to the industrial environment. The proposed architecture is organized in layers and it exploits the performances of the data processing at the edge of the network to reduce the bandwidth used for the cloud connection but also to obtain a shorter response time when dealing with time-critical operations. Fog/edge computing can integrate features such as low latency, predictability, hard real-time that are very important for monitoring and controlling time-critical operations specific to the industrial environment. Furthermore, the architecture contains a virtual environment used for interaction and data exchange between the virtual and/or real objects/things. Unlike the IIoT architectures presented in the specialized literature, the proposed IIoT architecture aims to integrate many fieldbuses into the system. In addition, by using a device description language, they can be added to the system through the plug and play method.

The proposal and description of complete IIoT software represents the main contribution of this paper. The conceptual model for the IIoT system contains some operational and implementation details that are useful in developing an IIoT solution based on the proposed architecture. The strengths of the proposed architecture are:

- Versatility—it can be adapted to the requirements and configuration of different industrial environments;
- Real-time capabilities—using System on Chip (SoC) systems with specialized processors for the design and development of the fog nodes;
- The integration of several fieldbuses at the same time;
- Interoperability between different fieldbuses by building a unified address space;
- Interoperability between IIoT systems by using a standardized middleware system.

This paper is structured as follows: Section 2 presents some IIoT architectures presented in the specialized literature and some companies, and the motivation of the proposed IIoT architecture. Section 3 describes the proposed IIoT architecture with some discussions related the implementation. Section 4 presents some discussions related to the security issues addressed by the proposed IIoT architecture. Section 5 presents a comparison between proposed architecture and the architectures analyzed in Section 2. The conclusions are drawn in Section 6.

## 2. Related Work

An IIoT reference architecture is the abstractization of an IIoT architecture that allows the identification of general components and implementation challenges. In the specialized literature, more reference architectures are proposed for IIoT. In June 2019, the Industrial Internet Consortium (IIC) released version 1.9 of the reference architecture for the IIoT [24]. It is focused on different viewpoints (business, usage, functional, and implementation viewpoints). The implementation viewpoint presents the system components and the technologies that can be used to implement the functionalities described in the functional viewpoint. Furthermore, the implementation viewpoint contains a technical description of the architecture components, including interfaces, protocols, behaviors, and other properties [24], but it does not contain information related to the integration of the fieldbuses and devices from the industrial environment.

Another important IIoT reference architecture is the Reference Architectural Model Industry 4.0 (RAMI 4.0) [25,26]. This is based on a 3-D model where the axes are Live Cycle, Value Stream, and Hierarchy Levels, and it is service-oriented architecture. The hierarchy level of the architecture is organized in the following levels: product, field devices, control devices, station, work centers, enterprise, and connected world. The value stream level of the architecture is related to the different component functionalities, it includes a communication layer and it is organized the following layers with a high level of abstraction: asset, integration, communication, information, functional, and business.

Other IoT reference architectures are organized on layers, such that defined by the International Telecommunication Union (ITU) that consists of four layers: devices, network, service support and application support, and application [27]. The device layer includes the device capabilities for interaction for the communication network and gateway capabilities for supporting multiple communication networks. The network layer includes networking and transport capabilities, service support, and the application support layer includes the generic and specific support for the application layer.

In [28], the authors proposed a case study on the growth of big data in IIoT systems, a classification of key concepts, and a presentation of key frameworks and continued with the presentation of future technologies, opportunities, and challenges. These researches concluded that augmented reality, IoT devices, cyber-physical systems and Industry 4.0 Big Data and Analytics (BDA) platforms are at an early stage in IIoT systems and that solutions should be found in developing new standards that allow interoperability between various platforms but also processing capability of the end-to-end applications for concentric computing systems. A solution to improve the blockchain scalability of IIoT systems by guaranteeing system security, latency but also decentralization, that was proposed in the article [29] have led to performance optimization with a deep reinforcement learning technique (DRL). The authors obtained results that demonstrated they can obtain a better efficiency compared to the basic parameters of the system.

In another research paper [19], the authors concluded based on conducted researches that the success of IIoT can be hindered for different reasons such as challenging collaboration between various heterogeneous IIoT systems, efficient data management, large, solid, and flexible data technologies, IIoT protocols, operating systems, reliable IIoT systems but also the coexistence of wireless technologies.

In addition to a survey of existing definitions of IIoT in [30], the authors also present a proposal for a new definition of what the IIoT means. The authors also present an analysis of a framework for IIoT devices based on security-related issues surrounding IIoT but as well as an analysis of the relationships between cyber-physical systems and Industry 4.0.

A secure fog-based IIoT architecture by suitably plugging a number of security features that reduce the trust and burden on the cloud and resource-constrained devices, but also that reduce latency in decision making that improves the performance, is presented in another research paper from the specialized literature [31]. Also, the authors demonstrated that by offloading several computationally intensive tasks to the fog nodes, the battery life of the resource-constrained of end devices is greatly saved. The validation of the architecture was demonstrated through theoretical analyses, practical experiments, but also through simulation and testbed implementation.

In [32], an IIoT application for a sewage treatment plant is proposed. This IIoT solution uses control station systems based on the STMicroelectronics STM32 microcontrollers to update the automation system and to activate the IIoT concept. Basically, the STM32-based control stations act as gateways between field devices connected to fieldbuses and their connection within the local network and further to the Internet for remote monitoring and cloud connections. The monitoring operations can be performed remotely from PCs or mobile devices such as smartphones and tablets. The authors concluded that this solution provide the real-time performances and reliability required for monitoring and controlling the sewage treatment plant.

In [33], the authors define an architecture organized on four layers: sensing layer, networking layer, service layer, and interface layer. In [34] an architecture is proposed that contains the physical layer, transport layer middleware layer, and application. An architecture with three layers (perception/sensing layer, network layer, and service/application layer) is proposed in [35,36]. All these reference architectures have a high level of abstraction and do not provide details related to the development of the IIoT solutions based on these reference architectures.

A trust framework for automated guided vehicles (AGVs) in smart factories is presented in [37]. The authors defined a trust measure for AVG, designed the trust framework, and presented a set of experiments based on this framework, but they did not provide details related to the communication with the AVGs and how the data are acquired from the AVGs and integrated into the proposed framework.

A software architecture for IIoT is presented in [38] and updated in [39]. The proposed architecture is organized on 4 levels: things, data provider, middleware, and applications. The interaction between things is performed at the application level, the upper level in the architecture. In this paper, we propose an evolution of the architecture presented in [38,39] by including new technologies such as fog/edge computing concepts. Furthermore, now things interact with each other at a lower layer, and at the application/service layer the services necessary for the development of applications are provided, such as monitoring and control specific to the industrial environment or applications for manufacturing tracing and monitoring. In addition, the proposed architecture provides implementation details, not being an abstract architecture as are most of reference architectures from the specialized literature.

Regarding SoC systems that can be used for IoT and IIoT, in [40] the authors present a comparison between several single-board computers that can be used for IIoT solutions, namely Raspberry Pi 3, BeagleBone Black, Banana Pi, and ODROID-C1. Of these, the most popular system is the Raspberry Pi, but in terms of the peripherals provided, the BeagleBone Black is the best. In [41], the authors propose a SoC architecture for the Industrial Internet of Things. The SoC architecture is implemented on an Field Programmable Gate Array (FPGA) system with two ARM processors that execute the Linux operating system. The FPGA gates are used to implement real-time operations specific to the industrial environment.

A detailed survey related to the security in IIoT is presented in [42], where the authors performed a systematic review of the literature and identified the security requirements for IIoT. These security requirements are related to the confidentiality, integrity, availability (CIA) triad, authentication, access control, maintainability, resilience, data security and data sharing, security monitoring, and network security. Additionally, the authors included a description of how fog computing can address these requirements and identify some research opportunity for the use of the fog computing for IIoT security, namely fog-enabled authentication, fog-enabled access control, fog-enabled maintainability, fog-enabled resilience, and fog-enabled data security and data sharing.

A survey for the security of the IIoT protocols is presented in [43]. They focused on the main 33 protocols and standards used in IIoT (from middleware systems to fieldbuses) and identify the security vulnerability with the common vulnerability scoring system (CVSS). They proposed a vulnerability analysis framework (VAF) that can be used to analyze 1363 vulnerabilities for IIoT systems. Data security in edge-based IIoT is investigated in [44]. Authors identify four main challenges reliable data storage, convenient data usage, efficient data search, and secure data deletion, and they proposed a cloud-fog-edge device storage framework for IIoT that addresses the challenges identified.

## 3. Description of the Proposed IIoT Architecture

In this section, we propose an IIoT software architecture that can be used in the development of the practical IIoT solutions. Before describing the proposed architecture, we will discuss the main challenges in the design and development of the IIoT architectures. The main requirements for the development of an IIoT architecture are represented by modularity, scalability, and interoperability between the different technologies used in the industrial environment. While IoT is human-centered, IIoT is machine-oriented [4] and is based on existing technologies and devices for reliability.

The IIoT systems are a special class of IoT systems that have additional requirements related to latency, real-time capabilities, security, reliability, and safety. These requirements result from the specific nature of the industrial environment where time-critical activities can be carried out. Perhaps the most important challenge in developing an IIoT architecture is data security because most data in the industrial environment are not public. For this reason, there must be a clear policy regarding restricted access to data only from trusted entities.

Another challenge in developing an IIoT system is the large number of industrial networks (fieldbuses) used in the industrial environment, each with its own characteristics related to the latency, real-time capabilities, transmission rate, and line protocol. All these fieldbuses should coexist in an IIoT system. Furthermore, in the industrial environment, there are many heterogeneous systems (ex. devices, sensors, programmable logic controllers, and human-machine interface) that must collaborate within an IIoT system. The heterogeneous nature of the systems results from the data source data, data stream, data storage, and processing requirements. Another challenge is the performance related to soft and hard real-time capabilities. Usually, in the industrial environment, critical activities are carried out for missions and safety with imposed requirements regarding timing and reliability. The IIoT architecture proposed in this article addresses all these challenges.

**Figure 1:**
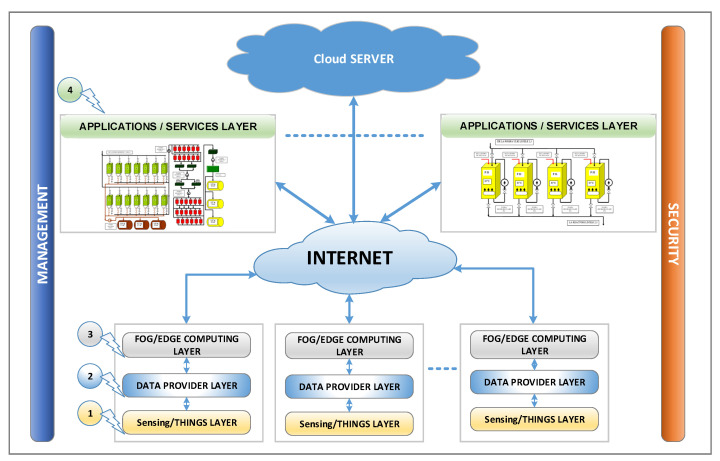
The proposed Industrial Internet of Things (IIoT) architecture. 1 Sensing/things layer, 2 Data provider layer, 3 Fog/edge computing layer, 4 Applications/services layer.

The proposed architecture is organized on layers and includes the edge/fog computing paradigm applied for the IIoT and support for the real-time and low latency requirements specific to the industrial field. Figure 1 presents the proposed software architecture organized on four layers: sensing/things, data provider, fog computing, and applications/service. In addition, fog computing and application/service layers can be connected to a cloud server to retrieve and save data. Each layer includes services for management and security through which the user can configure the functionality and security implementation at the layer-level. In Figure 1 the management and security capabilities are presented vertically because they transcend all layers of architecture.

**Figure 2:**
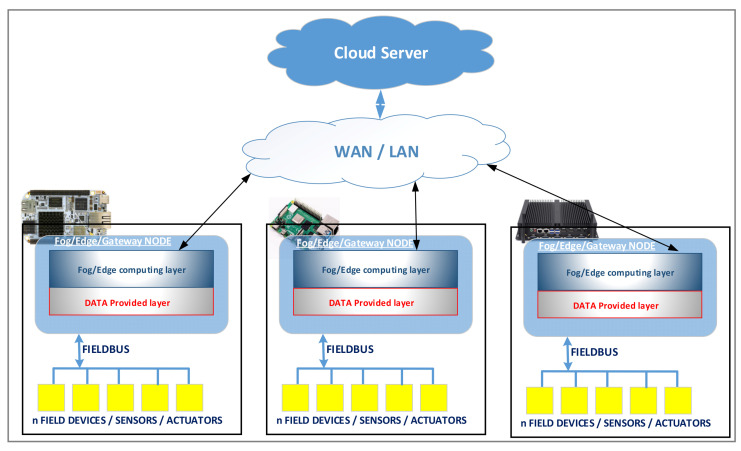
Other perspective of the proposed IIoT architecture based on fog/edge/gateway nodes.

An important aspect of the proposed architecture is the integration of fog/edge computing concepts. Thus, in the proposed architecture there are fog/edge/gateway nodes that integrate the fog/edge and data provider layers presented in Figure 1. There may be more such nodes in the IIoT architecture implementation. In this context of fog/edge/gateway nodes, another perspective of the proposed architecture is presented in Figure 2 (without application/service layer). In the IIoT architecture, there can be several nodes, each node connects to one or more fieldbuses, and these nodes implement the fog and data provider layers for these fieldbuses. These layers are software packages implemented for a specific device based on a microprocessor or a SoC (System on Chip) that runs on a high-level operating system such as Windows, Linux, or Android. These devices have peripherals such as USB (universal serial bus), CAN (controller area network) bus, UART (universal asynchronous receiver-transmitter), SPI (serial peripheral interface), and I2C (inter-integrated circuit) that allows the interfacing with fieldbuses used in the industrial environment. For example, through USB we can connect to CANOpen fieldbus using a USB-CAN interface, the CAN bus can be used to connect to CANOpen fieldbus, the UART can be used to connect to Profibus or Modbus fieldbus via a UART-RS485 interface, and SPI/I2C can be used to connect to CANOpen fieldbus via an SPI-CAN or I2C-CAN interface.

These fog/edge/gateway nodes can be implemented on various computing systems such as BeagleBone AI, Raspberry PI 4, industrial PCs or other types of computing system that allow connection to industrial networks and sufficient computing power for data processing. For the Internet connection, 5G modules can also be used to have a high bandwidth regardless of the physical location of the node. The 5G is a technology that is beginning to be adopted by most GSM (Global System for Mobile Communications) service providers. The 5G modules are the hardware support for the Internet connection and, for this reason, we will not insist on this aspect. From the software point of view, how the connection to the Internet is made is transparent. The following sections will be described in detail for each layer of the four layers in which the proposed architecture is organized.

### 3.1. Sensing/THINGS Layer

At this level, there are physical devices, sensors, actuators, and PLCs (programmable logic controllers) specific to the industrial environment. These devices are connected to a specific fieldbus and can send and/or receive data from this fieldbus. Examples of such fieldbuses are CANOpen, Modbus, Profibus, Profinet, EtherCAT, and WirelessHART.

At the upper layer, there must be different adapters/interfaces for communication with devices connected to fieldbuses. For example, USB-CAN interfaces or SoC-based systems with a CAN port can be used for a CANOpen fieldbus. Also, at the upper layer, there must be extensions through which the devices connected to the network can be configured, depending on the characteristics of each fieldbus. Due to the nature of the industrial environment, the complexity, and the requirements for the execution of time-critical activities, some configuration operations of the field devices must be performed manually. Once this operation is performed, the fieldbus can operate without major configuration changes. The purpose of this architecture is not to go into detail, it is considered that the networks are configured and at the top layer drivers specific to each fieldbus and the SoC on which the fog/edge/gateway node is implemented can be designed and developed. This layer is defined in the proposed IIoT architecture only from the hardware point of view, from the software point of view, at the upper level there must be modules through which the fieldbuses and devices connected to these fieldbuses can be configured.

### 3.2. Data Provider Layer

The purpose of this layer is to acquire data from devices connected to fieldbuses and store it in a buffer memory in order to be transmitted to the next layer or to send data from the memory buffer to the fieldbuses when data is received from the upper layer. It must be specified that the data flow is bidirectional, in the sense that the values from different sensors/devices can be acquired from the fieldbuses or commands that can be sent to the actuators connected on the fieldbuses.

As we specified previously, this layer together with the fog/edge layer is designed and developed to be executed on fog/edge nodes. These nodes can be represented by PCs or SoC-based computing systems that have features and can help to achieve real-time and reliable communication with fieldbuses. A system with sufficient computing power to process the data and peripherals needed to connect to the fieldbus that is monitored by the fog node must be chosen.

**Figure 3:**
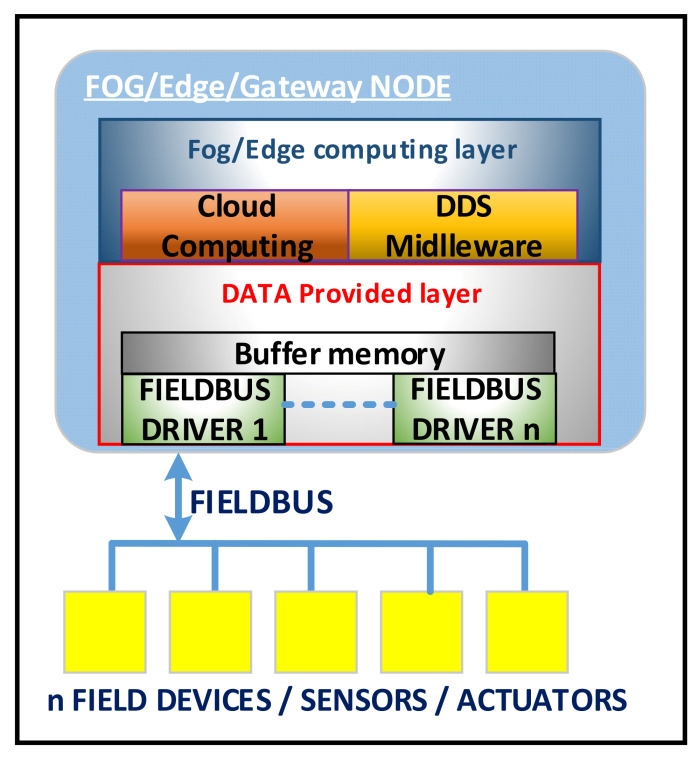
Software architecture of a fog/edge/gateway node.

The software architecture of a fog/edge/gateway node is presented in Figure 3. It can be observed from Figure 3 that the data provider level connects directly to the fieldbuses, and a fog/edge node can connect to one or more fieldbuses depending on the IIoT application and connection capabilities of the fog/edge node. Due to the diversity of fieldbuses that can be used in the industrial environment, this layer contains a driver for each type of fieldbus that can be integrated into the proposed IIoT architecture. These drivers implement the fieldbus protocol stacks for which it is designed and developed, and the connection method/way for fieldbuses must be considered, using an interface or directly throughout a peripheral (e.g., for a CANOpen fieldbus we can use a CAN port, a CAN-Ethernet interface, or a CAN-USB interface, depending on the connectivity provided by the SOC system used). From Figure 3, we can see that the data provider layer includes the drivers for the fieldbuses. A node can instantiate one or more drivers depending on the capabilities of the fog system and the configuration of the industrial environment that is included in the virtual industrial environment internet of things. We can design and develop drivers for mature networks that exist in the industrial environment for a long time or on new communication systems such as Narrowband IoT or LTE Cat-M based on 5G technology.

In order to meet the real-time requirements specific to the industrial environment, the fog nodes can be designed and developed on SoC systems with specialized coprocessors for the communication implementation with fieldbuses. For example, we can use SoC systems that have one or more cores based on the ARM Cortex Ax architecture for the execution of the complex operations and specialized processors (e.g., based on ARM Cortex Mx architectures or other optimized architecture as proposed in [45]) for the communication in real-time with the fieldbuses. Thus, the tasks with specific real-time requirements for time-critical operations specific to the industrial environment are executed on the specialized coprocessor where these requirements can be fulfilled and guaranteed. The basic idea is to use multiprocessor SoC systems that have processors on which Linux Embedded can be executed for the execution of non-critical operations in the best-effort (soft real-time) manner and specialized coprocessors on which a real-time operating system [46] can be executed for the implementation of the protocol stack and the execution of critical time operations. With this solution, low and predictable latencies can be achieved at the occurrence of events or at the execution of the critical operations by decoupling the software part with real-time requirements from the software part that does not have strict real-time requirements. In the case of these SoC systems, the drivers have a part that runs on specialized coprocessors (real-time cores) and a part that runs on the main processors (application cores). The software part from the specialized coprocessors implements the protocol stack, and the software part from the main processors works as a wrapper that saves the unit data in a buffer memory from where it will be taken and processed by other software modules. In terms of implementation, this solution involves the development of software for asymmetric multi-processing, and great care must be taken at the implementation of the communication between processors in order not to introduce performance bottlenecks. Fog nodes can also be developed on computing systems without specialized co-processors, but in this case, we can obtain at most soft real-time capabilities due to the limitations of Windows and Linux operating systems [47] (including real-time extensions such as RTAI (Real-Time Application Interface) or PREEMPT_RT). The specialized co-processors provide support for the development of small software modules with strict hard real-time capabilities and predictable latencies.

**Figure 4:**
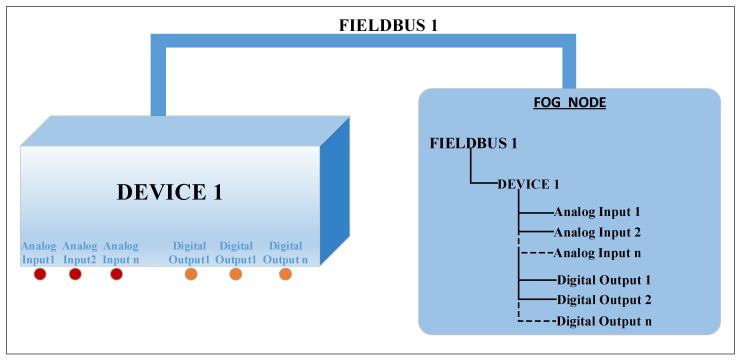
Example of the address space for a device with n analog inputs and n digital outputs.

Each fieldbus will have an associated address space that defines the things connected to the fieldbus. One of the main goals of the drivers is to build the address space in a unitary way, hiding the specific details of each fieldbus and connected devices, such as the addressing mode or the message format. Thus, it is possible to obtain the aggregation and processing of the data acquired from different fieldbuses to which the fog node is connected through a unique software interface, ensuring interoperability between different fieldbuses. The structure of this address space is organized in a tree and it is stored in extensible markup language (XML) files that are loaded when the software module that implements the data provider layer is instantiated. It is considered that a fieldbus can have several devices/sensors/actuators connected, and each device can have several things such as a temperature, a pressure, a level, and so on. Each thing is characterized by several attributes such as data type, quality, timestamp, and access type. The data type can be numeric, string, and logic, and the quality can be good or bad. The timestamp represents the time and date of the last update of the things, and the access type can be read-only, write-only, and read-write. A sensor will have the read-only access type and an actuator can have write-only or write-read access type. An example of a space address for a device is presented in Figure 4. Thus, we have a device that has n analog inputs and n digital outputs. Analog inputs can be connected to sensors such as thermocouples, a resistance thermometer, pressure transducers, etc. and digital outputs that can be connected to execution elements via relays. The address space for this device built in the fog node contains n things for the n analog inputs and n things for the n digital outputs, as illustrated in Figure 4.

The data acquired from the fieldbuses are saved in a buffer memory, where they will be retrieved and transmitted to the upper layer. The buffer memory is allocated according to the definition found in the XML configuration files associated with each fieldbus. The user has the possibility to configure the communication parameters on the network as well as the introduction or elimination of new devices. This address space is used by the upper layer to read/transmit data to the thing. A thing can be accessed through a path in the form of “network name. device name. thing name”. Each device will have associated an XML file that defines all the things and attributes of the things it makes available. These files contain generic names for things that can be replaced with aliases in the network setup process. Each fieldbus will have an associated XML file in which network-specific configurations (such as communication speed) and the list of devices connected to the fieldbus along with the associated XML files are stored. A fog/edge node has an associated XML file in which the list of fieldbuses to which it connects with the associated XML files are stored. These files are automatically updated during the configuration of fieldbuses to which the fog node connects and loaded when the software module that implements the functionalities of the fog node is executed.

An important part of the XML file associated with a device is the part that describes how the value of a thing is read or written. This is translated into messages that can be transmitted and read on the fieldbuses, depending on the characteristics of each network. This means that the XML file must also be interpreted by the driver to take over the commands/messages specific to each thing.

Depending on the type of network, an acquisition cycle can be defined by which data in the buffer are updated periodically and prioritized depending on the criticality of the monitored data and the user configuration. An example of such an acquisition cycle is presented in [48].

### 3.3. Fog/Edge Computing Layer

**Figure 5:**
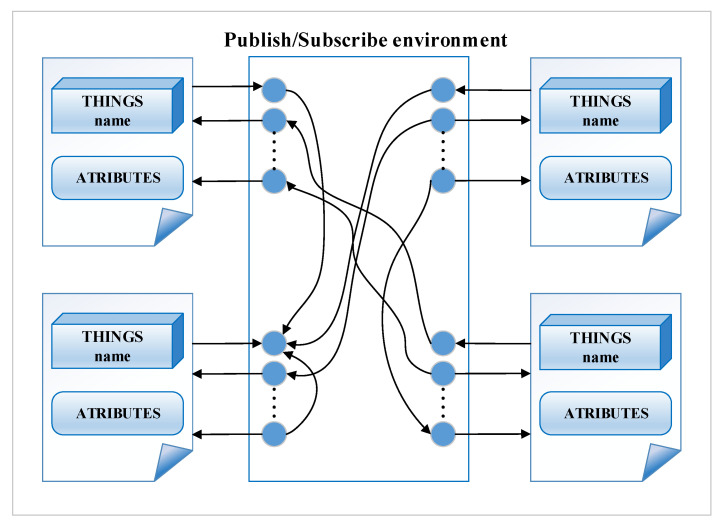
Interactions between things in a publisher-subscriber communication environment.

At this layer, the local processing of the data acquired from the industrial fieldbuses is performed and it is the place where things interact with each other. Practically at this level, the operations specific to the edge and fog computing concepts are performed. Thus, in order to interact between the things, it is proposed to use the publisher-subscriber paradigm, in the sense that a thing publishes its value periodically or when an event occurs, and other things subscribe to this value. There are two types of thing: physical things that are associated with things defined at the data provider layer and virtual things that can be defined at this level and represent the result of processing data from physical and/or virtual things. The virtual things subscribe to one or more values published by the other things, perform certain processing on the read values (mathematical operations, mathematical functions, aggregation functions, etc.) and the obtained result is published as a new value. From the functional point of view, no distinction is made between virtual things and physical things. Things can connect to one or more things in a virtual communication environment that is based on the publish-subscribe paradigm and that performs some processing on data and can publish its value. A graphical representation of the virtual publishing-subscriber environment is shown in Figure 5. Some things only publish data, for example, things associated with a sensor such as temperature sensor, pressure sensor, and things that subscribe to one or more values but do not publish any values, such as things that are associated with actuators from the industrial environment. Internally, things perform a wide range of data processing tasks such as mathematical operations, mediation, aggregation, or other mathematical functions. There may be cloud things that subscribe to certain data and save it to a remote or local cloud server, or things that connect to data and save it to a remote or local database. These types of thing perform certain cloud services on the fog node, such as storage services.

The interaction between things can be undertaken at the level of a fog/edge node, but we also want the interaction between things from different fog/edge nodes. In order to achieve requirements related to the interaction between things, a middleware system that provides support for publish-subscribe communication can be used. At the moment, there are several middleware systems that operate on the publisher-subscriber paradigm, such as the advanced message-queuing protocol (AMQP) [49], message-queuing telemetry transport (MQTT) [50], and data distribution service (DDS) for real-time systems [51]. We focused on middleware systems that are based on the publish-subscribe paradigm because the IIoT system will be much more versatile. There are other important middleware systems that are widely used in the development of IoT systems [52] such as XMPP (extensible messaging and presence protocol) or CoAP (constrained application protocol) but these middleware systems have not been considered because they are not based on the publish-subscribe paradigm. From the middleware systems that are based on the publish-subscribe paradigm, we suggest the use of DDS for real-time systems is chosen because it was designed specifically for real-time systems. In order to argue for the selection of the DDS protocol, the study by [52] can be analyzed, where the HTTP (S), CoAP, MQTT, AMQP, XMPP, and DDS are identified as the main middleware systems based on messages that can be used in the development of the IoT systems. From these middleware systems, MQTT and AMQP use a broker that can be a bottleneck point. The DDS is the only one that uses a bus-like architecture (MQTT uses tree architecture and AMQT uses star architecture), has 23 quality of service (QoS) levels (MQTT and AMQP uses 3 QoS levels), is data-centric, and implements a security mechanism like TLS, DTLS, and DDS Security. Also, the data distribution is very versatile with the possibility to achieve 1 to 1, 1 to N, and N to 1 [52]. By using a standard middleware system, interoperability can be ensured between the IIoT systems and with other systems could take data from the IIoT system.

Thus, a DDS domain is defined for the architecture, which is divided into several DDS partitions, a DDS partition corresponding to a fog/edge node. DDS defines a unit of information as a topic. Thus, for each thing defined at the data provider level, a topic will be associated, which will have the unique name of the access path defined at the previous level. In addition to these things, virtual things are also defined, things that can subscribe to different DDS topics, and different processing is performed on the received data. All these things are created by the user in the configuration process based on predefined things that are instantiated and that subscribe to one or more topics. Perhaps the most important predefined virtual thing is the expression thing in which certain mathematical operations and functions can be applied to the data with which it is subscribed. Thus, the user can create virtual things according to his needs and in accordance with the application of the industrial environment that is integrated into the IIoT architecture. Due to the solution adopted for the interaction between things, the architecture is very versatile, being able to be used in a wide range of industrial applications.

**Figure 6:**
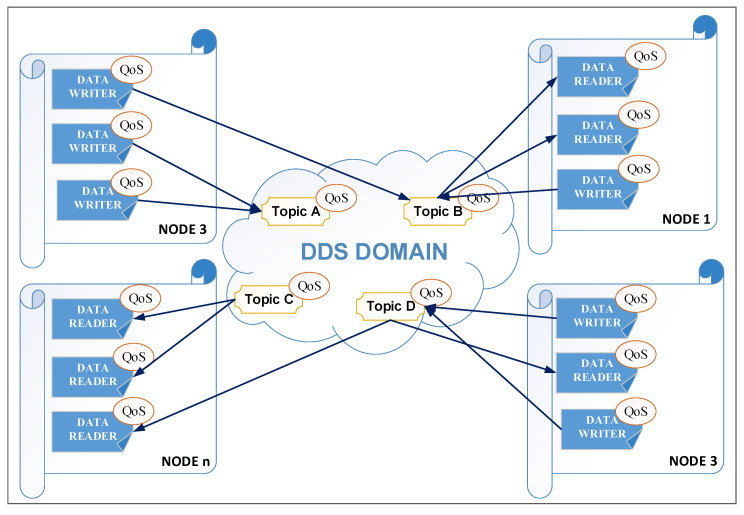
Interactions between things in a data distribution service (DDS) domain.

In the case of using the DDS middleware system, the publishing-subscription environment from Figure 5 can be represented as in Figure 6. The connections between things are not made only locally, but in the DDS domain, which is defined at the level of the IIoT system, things from the DDS domain can be from the same node or from remote fog/edge nodes in a local network or on the Internet.

As can be seen from Figure 6, in the case of DDS middleware, each topic is associated with different QoS policies. These policies can be related to data availability, data delivery, data timeliness, and resources. For data availability, we can control how data is available within a domain through the DURABILITY, LIFESPAN, and HISTORY policies. For data delivery, we can control how data is delivered and exclusive rights for data through the PRESENTATION, RELIABILITY, PARTITION, DESTINATION_ORDER, and OWNERSHIP policies. For data timeliness, we can control the timeliness for the data through the DEADLINE, LATENCY_BUDGET, and TRANSPORT_PRIORITY policies. Through the resources policies, we can control the computing and network resources. All of these policies are described in the DDS specifications and they are included in the implementations of these specifications [51,52,53].

### 3.4. Applications/Services Layer

The application/services layer is the place where industry-specific applications can be developed, such as those for remote monitoring and control. We can develop a supervisory control and data acquisition (SCADA) type application by subscribing and publishing in the DDS domain where the things interact with each other. At this layer, it can be designed and developed software solutions/applications that can monitor things in the industrial environment and send commands to these things. Also, based on the data saved on the fog nodes or in the cloud, different reports and graphical representations can be made regarding the evolution of the data over time and of the industrial process. At this layer and depending on the capabilities of the fog nodes, on each fog node a web server can be developed that allows the visualization of the values published by the fog node in the DDS domain. The web server can also have an extension that provides the facility to configure the fog/edge node remotely, especially since fog/edge nodes can operate without the graphical interface.

At this layer, an HMI application can be designed and developed like that presented in [39] and which can be used to graphically visualization of data acquired from the industrial environment and send commands in the industrial environment through the graphical interface.

## 4. Security

Security is an important issue in any application distributed over the Internet. In the case of IIoT, it is even more important because non-public data is transmitted over the Internet, and access to this data must be granted on the basis of clear security policies. At the level of things/sensing, security is ensured by the capabilities of each fieldbus. Usually, in these fieldbuses, the security mechanisms are not implemented because not everyone has physical access to them. From a security point of view, the risks at this level are small, especially if physical access to them is restricted. In addition, devices connected to networks can have different security mechanisms in place. From the point of view of the current IIoT architecture, the security at this level depends on the configuration made at the level of each fieldbus and filed device used, and no new security mechanisms are implemented because this level does not involve the development of software modules.

At higher layers, the security mechanisms must be implemented at the fog node level. Remote access to fog nodes must be restricted, thus avoiding any security holes that may occur. The operating system must also be kept up to date with the latest security updates. Configuration XML files can be encrypted. This does not degrade the performance because they are decrypted when starting the software modules to implement the data provider and fog/edge computing layers, and they are saved in encrypted mode following the configuration operations performed by the user.

The biggest security issue can be when publishing data in the publisher-subscriber environment provided by the DDS domain defined for the current IIoT architecture because those messages can be intercepted when they are transmitted over the Internet. In 2018, the latest security specifications for the DDS protocol were published. These specifications are also implemented in the OpenDDS implementation, an open-source implementation that can be used for the design and development of fog/edge nodes. The specifications provide the plugins for authentication, access control, logging, data tagging, cryptography, and certificates [54]. Through these facilities, the data are transmitted encrypted over the Internet ensuring a high degree of security regarding confidentiality, integrity, and availability.

## 5. Discussions

Table 1 presents a comparison between the proposed architecture and those described in the related work section.

A more detailed quantitative or qualitative analysis between the proposed IIoT architecture and the existing ones requires more information than those presented in the literature. Moreover, we propose a conceptual model and the experimental results will be implemented and presented in future research articles.

For example, we can use BeagleBone Black for implementation. This is based on the Sitara™ AM335x SoC with 2 ARM Cortex A8 processors and two Programmable Real-Time Unit Subsystem and Industrial Communication SubSystems (PRU-ICSS). The ARM Cortex A8 processors use a Linux-based operating system. One PRU-ICSS can be used to design and implement a driver for the Modbus RTU (remote terminal unit) fieldbus and the other PRU-ICSS can be used to design and implement a driver for the CanOpen fieldbus. The data provider, fog computing, and applications/service layers can be designed and implemented to be executed on the ARM Cortex A8 processors under Linux. By using many of BeagleBone Black systems, we can build a complex IIoT system that can be geographically distributed.

## 6. Conclusions

This paper proposed a conceptual model software architecture for the IIoT. The proposed architecture is a conceptual model with a low level of abstraction, being oriented towards the design and development of real-time applications for the IIoT. Among the strengths of the proposed architecture, we can mention: the integration in the system of many fieldbuses used in the industrial environment, addressing the specific requirements of the industrial environment such as real-time and low-latency capabilities, and the integration in the architecture of the concepts of fog and edge computing. Also, the use of the SoC-based computing platform with specialized co-processors is proposed in order to be able to execute in real time certain time-critical operations specific to the industrial environment. The specialized co-processors provide support for the development of small software modules with strict hard real-time capabilities and predictable latencies while the main processors provide support for the development of software modules with soft real-time capabilities. We can conclude that the conceptual model is feasible for a combination of hard and soft real-time systems with hard real-time constraints provided by the specialized co-processors. The fog/edge computing can integrate features such as low latency, predictability, and hard real time that are very important for monitoring and controlling time-critical operations specific to the industrial environment. Furthermore, the architecture contains a virtual environment used for the interaction and data exchange between the virtual and/or real objects/things located on the same fog / edge node or on different fog/edge nodes. As future work, we intend to implement the proposed architecture on BeagleBone Black and BeagleBone AI systems.

## Figures and Tables

**Table 1:** A comparison between the proposed architecture and those described in Section 2.

	Implementation Details	Industrial IoT?	Organization	Fog/Edge Computing	Integrate Fieldbuses?
**IIC Reference Architecture [24]**	No. It is a reference architecture	Yes	Business, usage, functional and implementation viewpoints	Edge gateway	No details are given
**RAMI 4.0 [25,26]**	No. It is a reference architecture	Yes	3-D model with the axis: Cycle, Value Stream and Hierarchy Levels	No	No details are given
**ITU Reference Architecture [27]**	No. It is a reference architecture	No. It is for IoT	Four layers: devices, network, service support and application support, and application	No	No details are given
**Fog-based IIoT architecture [31]**	Details related the security on fog nodes	Yes	Four layers: perception, fog, cloud, application	Fog nodes	No details are given
**Sewage treatment plant [32]**	Details related the control stations based on a STM32 MCU and the use of the WeChatApplets	Yes	-	Gateways/control stations	Integrates field device without details regarding the communication interfaces
**IIoT software architecture proposed in [38,39]**	Details related to the middleware and application levels	Yes	Four levels: things, data provider, middleware, application	No	Modbus and CanOpen fieldbuses
**The proposed IIoT software architecture**	The use of the SoC-based computing platform with specialized co-processors in order for fog/edge nodes.	Yes	Four layers: sensing/things, data provider, fog/edge, applications/services	Fog/Edge nodes	Yes. It is proposed an unified address space and a driver for each field bus integrated
